# Impact of fractionated cisplatin and radiation treatment on cell growth and accumulation of DNA damage in two normal cell types differing in origin

**DOI:** 10.1038/s41598-023-39409-7

**Published:** 2023-09-09

**Authors:** Pamela Akuwudike, Milagrosa López-Riego, Cloé Dehours, Lovisa Lundholm, Andrzej Wojcik

**Affiliations:** 1 Centre for Radiation Protection Research, Department of Molecular Biosciences, The Wenner-Gren Institute, Stockholm University, Svante Arrhenius väg 20C, 106 91 Stockholm, Sweden; 2 Polytech Angers l École d’Ingénieurs, Angers, France; 3 Institute of Biology, Jan Kochanowski University, Kielce, Poland

**Keywords:** Chemotherapy, Radiotherapy

## Abstract

Evidence on the impact of chemotherapy on radiotherapy-induced second malignant neoplasms is controversial. We estimated how cisplatin modulates the in vitro response of two normal cell types to fractionated radiation. AHH-1 lymphoblasts and VH10 fibroblasts were irradiated at 1 Gy/fraction 5 and 3 times per week during 12 and 19 days, respectively, and simultaneously treated with 0.1, 0.2, 0.4, 0.8, 1.7 and 3.3 µM of cisplatin twice a week. Cell growth during treatment was monitored. Cell growth/cell death and endpoints related to accumulation of DNA damage and, thus, carcinogenesis, were studied up to 21 days post treatment in cells exposed to radiation and the lowest cisplatin doses. Radiation alone significantly reduced cell growth. The impact of cisplatin alone below 3.3 µM was minimal. Except the lowest dose of cisplatin in VH10 cells, cisplatin reduced the inhibitory effect of radiation on cell growth. Delayed cell death was highest in the combination groups while the accumulation of DNA damage did not reveal a clear pattern. In conclusion, fractionated, concomitant exposure to radiation and cisplatin reduces the inhibitory effect of radiation on cell proliferation of normal cells and does not potentiate delayed effects resulting from accumulation of DNA damage.

## Introduction

Chemoradiotherapy (CRT) achieves spatial cooperation via the locoregional control of tumors by radiotherapy (RT) and systemic control of metastasis by the use of chemotherapeutic (CT) agents^1,2^. In addition, the combination of RT and certain CT drugs has shown an enhanced eradication of the primary tumor^3,4^. Cis-diamminedichloroplatinum (II), commonly known as cisplatin or cDDP, is a common antitumor drug, frequently combined with RT^5–7^.

Improved cancer cure is associated with the risk of RT-induced second malignancies (SMN)^8^. The most common SMNs include soft tissue sarcoma, thyroid cancer, breast cancer and cancer of the central nervous system^9^. CT alone has also been implicated in the onset of therapy induced SMNs, especially acute myeloid leukemia (AML)^10,11^ in patients treated for pediatric cancers, ovarian cancers^11^, breast cancer^10^ and Hodgkin’s disease^12^. Although there is ample evidence associating RT alone or certain types of CT alone with SMN, there are uncertainties regarding the effect of combined treatment with both modalities. Results of the few epidemiological studies evaluating the effect of CRT on the incidence of RT-induced SMN are contradictory, but some indicate a reduction of the SMN risk which, for breast SMN, is interpreted as resulting from hormonal changes due to ovarian damage^13,14^. However, it is also conceivable to assume that CRT may reduce the risk of SMN due to excessive killing of damaged cells. In support of this, we recently observed a lower level of cytogenetic damage in lymphocytes of gynaecological patients receiving CRT as compared to RT alone that was associated with enhanced level of apoptosis in the CRT arm^15^.

Given the established application of CRT in tumour therapy^16^, a better understanding of combined effects is important. At the cellular level CT drugs may interact with RT via different mechanisms such as increased radiation damage, inhibition of DNA repair, cell-cycle synchronization, inhibition of pro-survival pathways and abrogation of rapidly dividing cells^17^. In order to shed more light on the mechanisms of interaction in normal cells, we have carried out experiments with AHH-1 lymphoblastoid cells and VH10 fibroblasts exposed to radiation and cDDP in fractionated setups with 1 Gy of ionising radiation (IR) given five times (AHH-1) or three times (VH10) per week and a range of cDDP doses (0.1–3.3 µM) given concomitantly two times per week. This exposure setup simulates concomitant CRT and is a novel in vitro study approach: previous combination studies analysed effects of single doses^18^. The analysed endpoints were selected to represent the mechanisms of CT and RT interaction discussed by Seiwert et al.^17^ The endpoints also reflect several hallmarks of cancer^19^. Cell growth was monitored during combined and single agent treatment in order to assess the impact on the ability of cells to proliferate while being treated. Immediate and long-term post-treatment effects were analysed in cells exposed to IR alone, the lowest dose of cDDP and the combination. The analyses included accumulated DNA damage indicative of carcinogenesis (gamma H2AX foci, micronuclei and giant cells), cell death (apoptosis in AHH-1 lymphoblastoid cells and senescence in VH10 fibroblasts), changes in radiosensitivity and stem cells markers. The study complements our earlier analysis of the impact of fractionated IR alone on AHH-1 and VH10 cells^20^.

## Results

**Figure 1:**
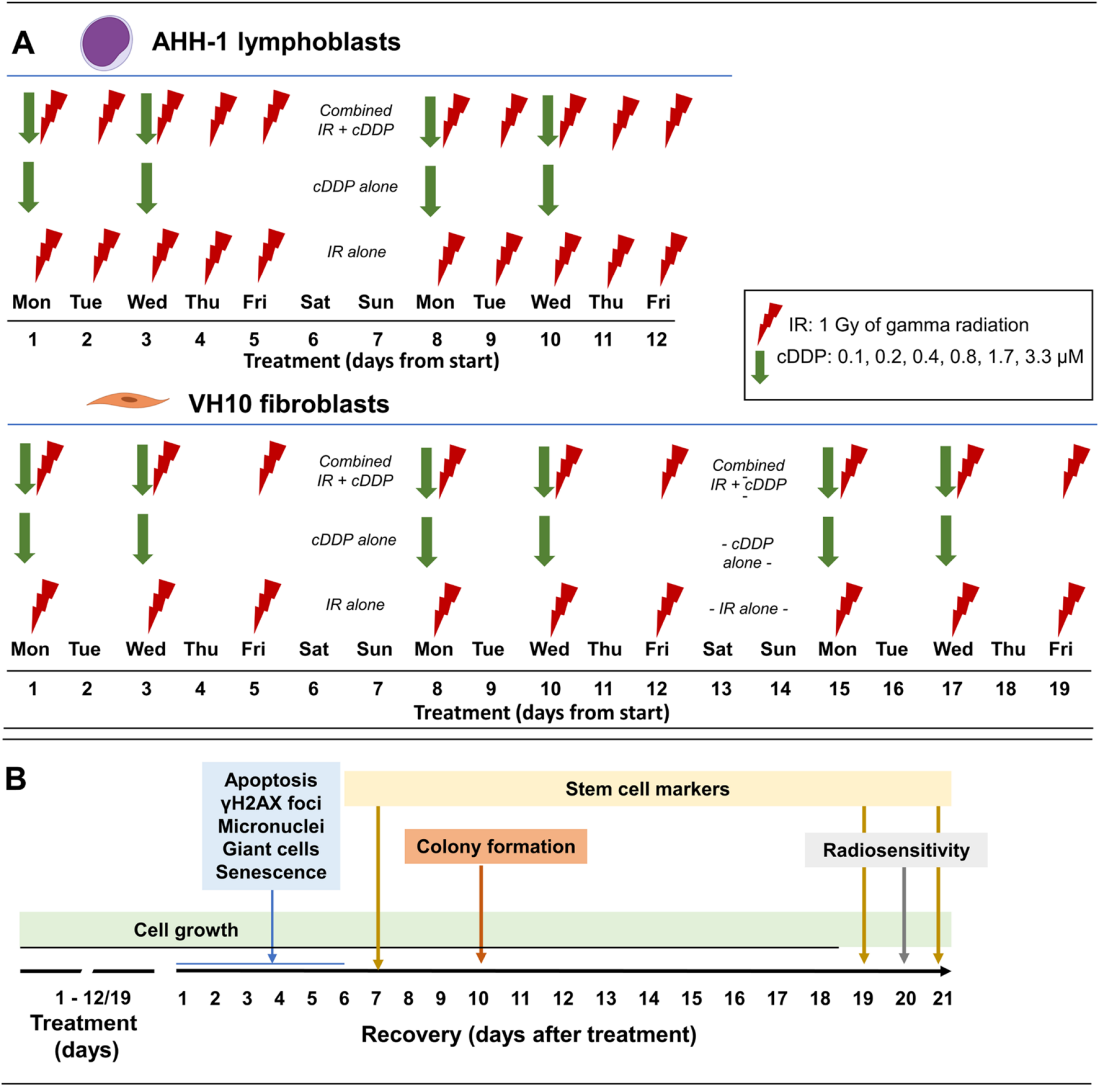
(**A**) Schematic representation of treatment scheme. (**B**) Schematic representation of experimental workflow after treatment. Vertical arrows in panel (**B**) show the day of analysis, colours correspond to the endpoint. *cDDP* cisplatin, *IR* ionizing radiation.

A schematic representation of the treatment scheme is shown in Fig. 1. The time points at which the post-treatment analyses were carried out were chosen to obtain meaningful information. Cell proliferation was analysed continuously. Cell death, γ-H2AX foci, micronuclei and giant cells were analysed at days 3 and 7 because the frequency of these events is expected to rapidly decline following termination of exposure. Colony formation was analysed on day 10 due to the very low attachment capacity of cells during the first week after fractionated exposure and therefore technical difficulty in performing the assay. Radiosensitivity and stem cells markers were assessed on day 21 to analyse the presence of long-term alterations in cellular properties. The expression of stem cell markers on day 21 was compared to values derived on day 7 in order to detect a possible kinetic of expression changes. The measured mode of cell death was selected to represent the dominating mechanism in cells of haematopoietic and epithelial origin, respectively: apoptosis in AHH-1 cells^21^ and senescence in VH10 cells^22^.

**Figure 2:**
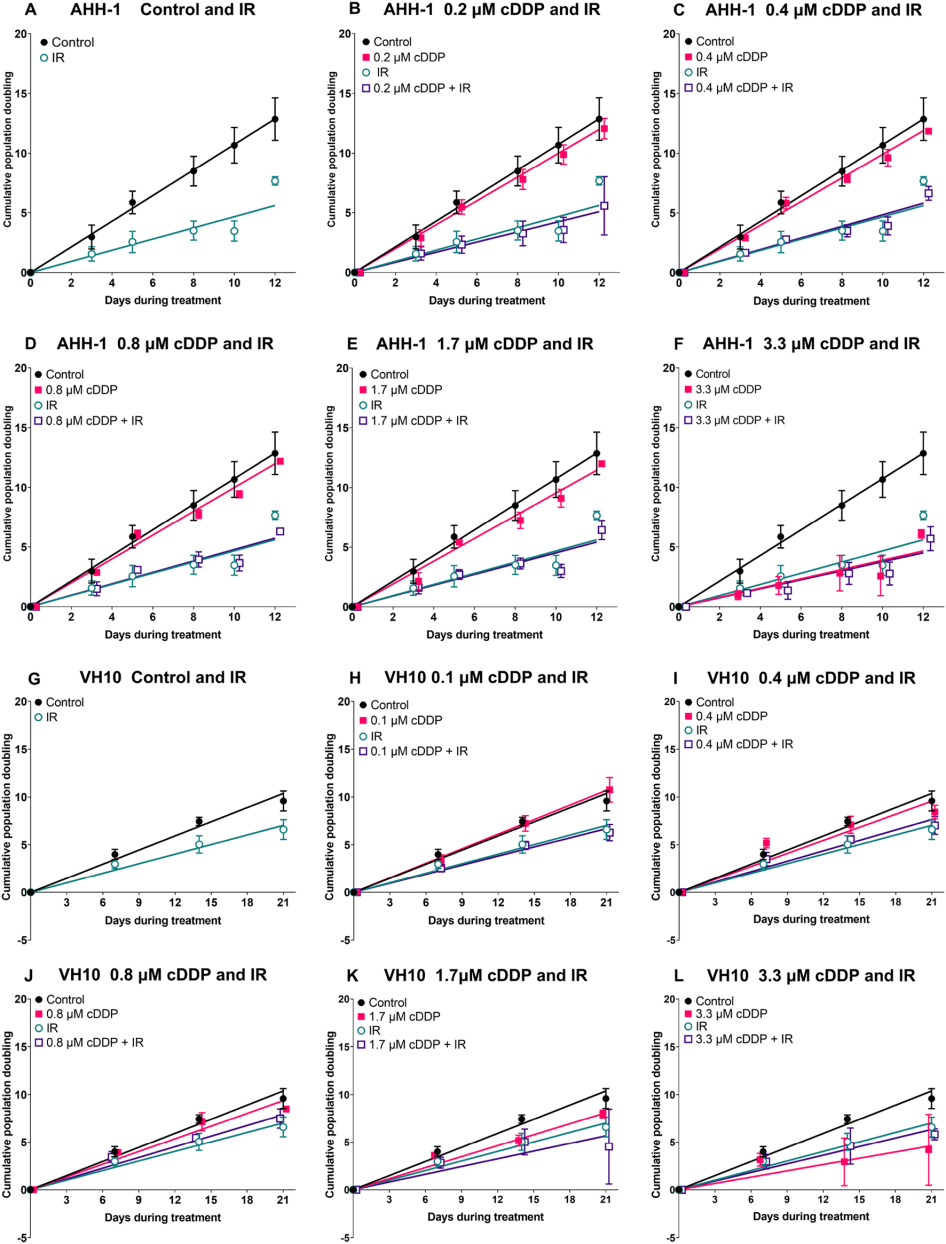
Cumulative population doubling growth curves of AHH-1 (**A**–**F**) and VH10 (**G**–**L**) cells during treatment. Curves were fitted to a linear equation and the slopes were compared using one-way ANOVA and Cohen’s effect size test. Error bars represent standard deviation from 3 independent experiments. P values and Cohen’s d values (effect size) are summarized in supplementary table S1. *cDDP* cisplatin, *IR* ionizing radiation.

**Figure 3:**
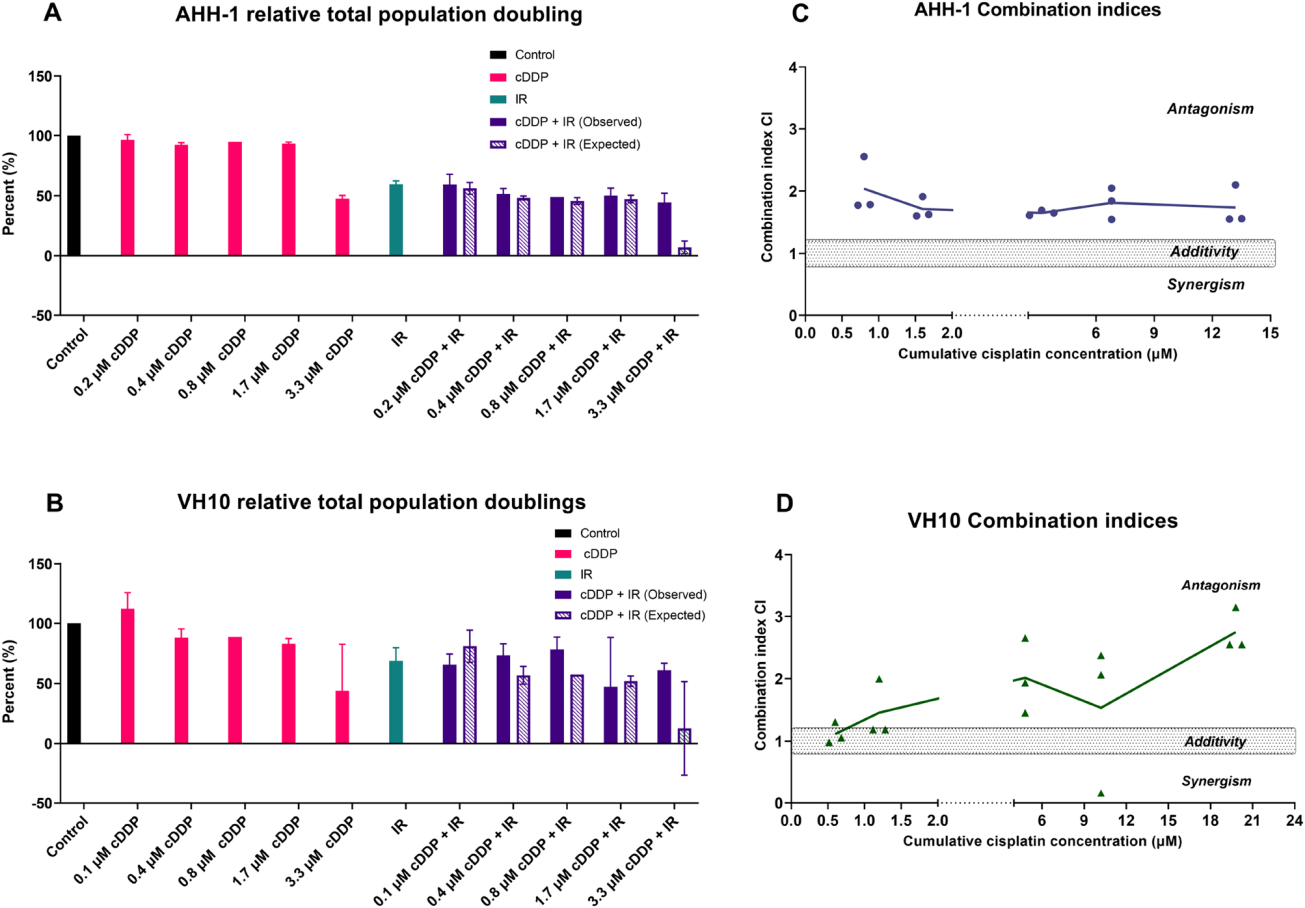
Interaction analysis of cDDP and IR. (**A**,**B**) Relative total observed and expected population doublings in AHH-1 and VH10 cells, cDDP doses are given per treatment. The expected values were calculated assuming additivity. Combination indices from each experiment are plotted as a function of total accumulated cDDP concentration in AHH-1 lymphoblasts (**C**) and VH10 fibroblasts (**D**). Solid lines represent the mean values. *cDDP* cisplatin, *IR* ionizing radiation.

**Figure 4:**
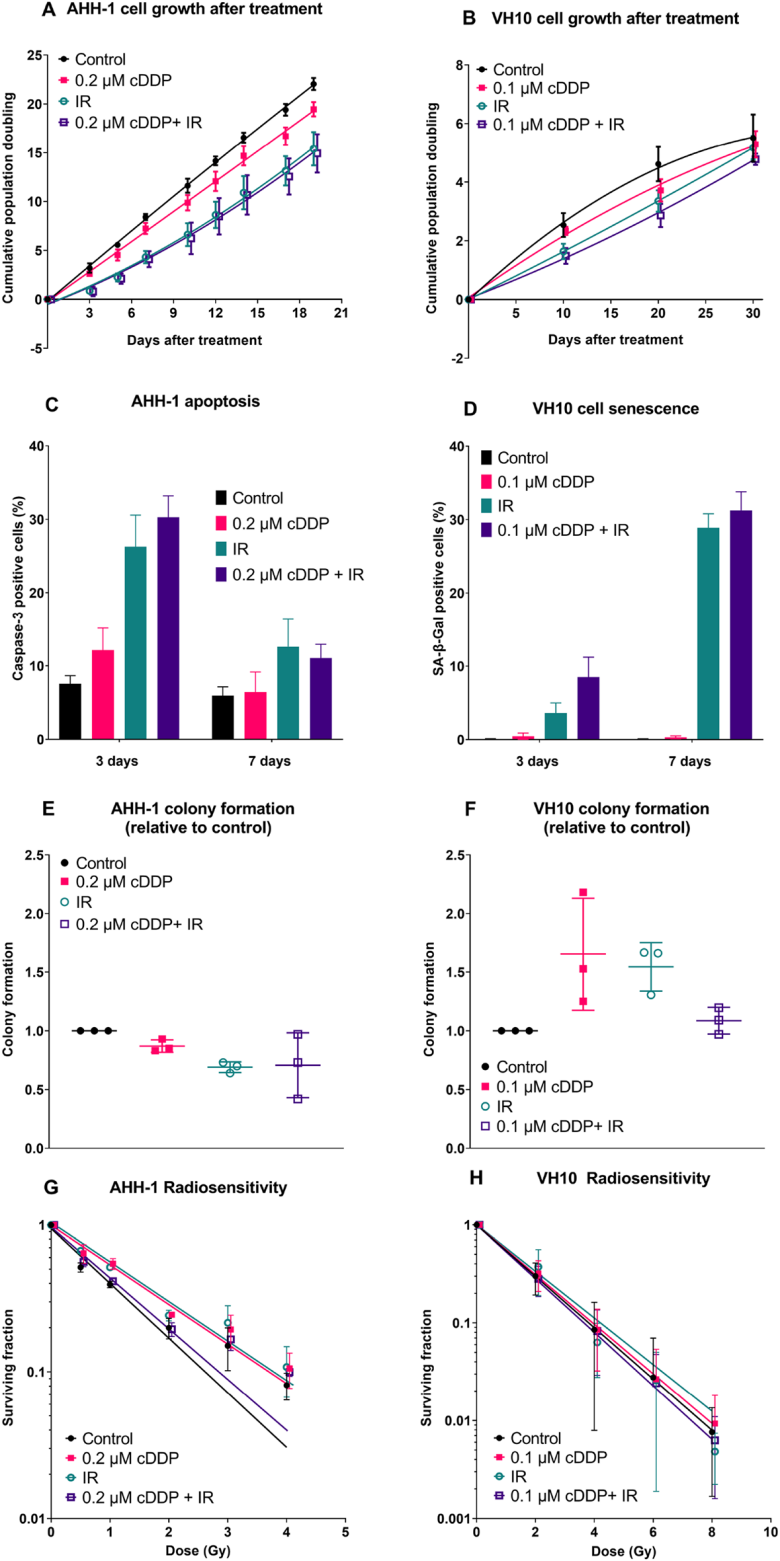
Effects of either cDDP alone, IR alone or combination measured after treatment. Cumulative cell growth after treatments of AHH-1 (**A**) and VH10 (**B**) cells. Cell death 3 and 7 days after treatment. Caspase-3 stained AHH-1 cells (**C**), and senescence-associated β-galactosidase stained VH10 cells (**D**). Colony formation 10 days after treatment in AHH-1 cells (**E**) and VH10 cells (**F**). Changes in radiosensitivity 21 days after treatment of AHH-1 (**G**) and VH10 (**H**). Error bars represent standard deviation from 3 independent experiments. P values (ANOVA) and Cohen’s d values (effect size) are summarized in supplementary table S2. *cDDP* cisplatin, *IR* ionizing radiation.

**Figure 5:**
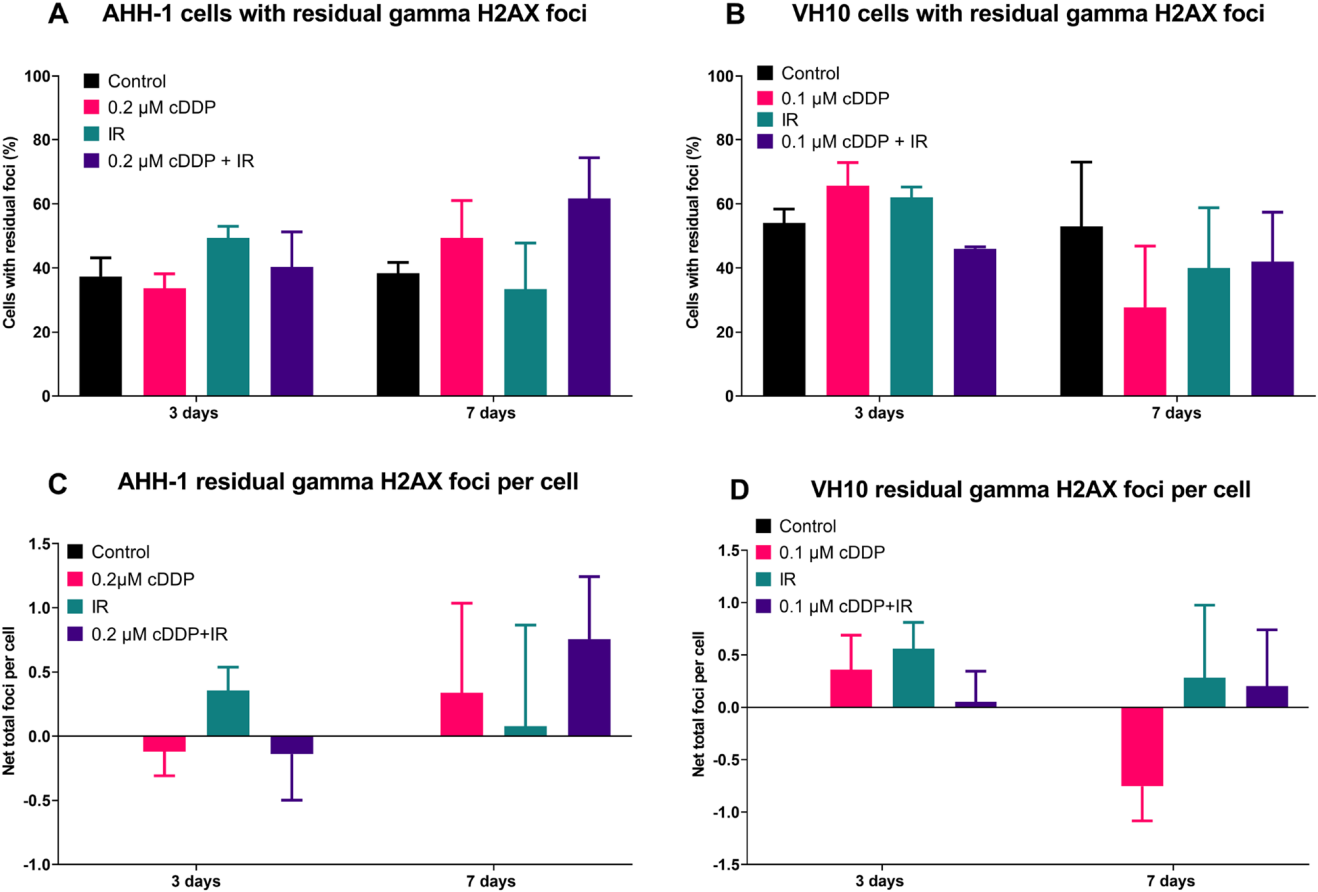
Accumulation of residual DNA damage after treatment. (**A**,**B**) Percent of cells with residual γ-H2AX foci 3 and 7 days after treatments. (**C**,**D**) Net focus frequencies (treatment minus control) per cell 3 and 7 days after treatment. Error bars represent standard deviation from 3 independent experiments. P values (ANOVA) and Cohen’s d values (effect size) are summarized in supplementary table S2. *cDDP* cisplatin, *IR* ionizing radiation.

**Figure 6:**
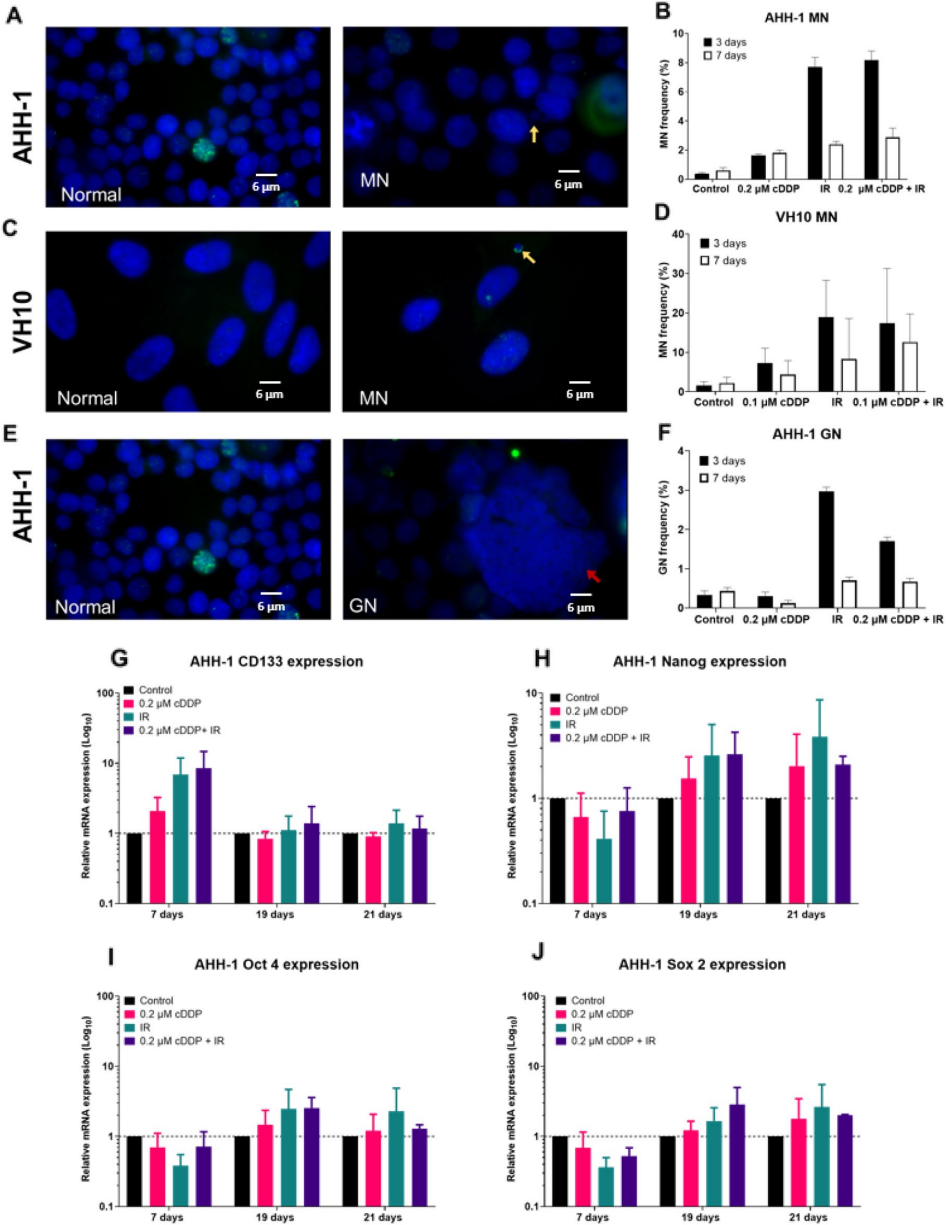
Accumulation of micronuclei (MN) and giant nuclei (GN) three and seven days after treatment. Fluorescence images (**A**) and frequency of MN (**B**) in AHH-1 cells. Fluorescence images (**C**) and frequency of MN (**D**) in VH10 cells. Fluorescence images (**E**) and frequency of GN (**F**) in AHH-1 cells. Cell nuclei were stained with DAPI (blue), green staining represents gamma H2AX. Gene expression of stem cell markers CD133 (**G**), Nanog (**H**), Oct4 (**I**), and Sox2 (**J**) in AHH-1 cells 7, 19, and 21 days after treatment. Yellow arrows on images indicate MN and GN. Error bars represent standard deviation from 3 independent experiments, except (**G**–**J**) 21 days, which are from 2 independent experiments. P values (ANOVA) and Cohen’s d values (effect size) are summarized in supplementary table S2. *cDDP* cisplatin, *IR* ionizing radiation.

All results are presented in Figs. 2, 3, 4, 5 and 6. Relevant P values (ANOVA) and d values (Cohen’s effect size test) of comparisons are given in supplementary tables S1 (for Fig. 2) and S2 (for Figs. 4, 5 and 6).

### Cell growth during treatment

Cell growth curves generated during fractionated treatment are shown in Fig. 2A–F for AHH-1 and Fig. 2G–L for VH10.

Fractionated IR significantly and very largely reduced cell proliferation in both cell types and, in accordance with survival results for acute exposure (Supplementary material, Supplementary results—differential sensitivity of AHH-1 and VH10 cells to cDDP and IR following single and fractionated exposure—Fig. S1), the effect was larger in AHH-1 than VH10 cells (Cohen’s d values 5.69 and 3.94, respectively). CDDP alone reduced the proliferation significantly only at the dose of 3.3 µM. 1.7 µM cDDP had no significant effect but reduced the growth of AHH-1 cells largely (d = 1.17) and of VH10 cells—very largely (d = 3.49). Combined exposure to IR and cDDP of both cell types resulted in growth curves that were similar to IR alone. The expected cumulative population doublings calculated based on assuming additivity between cDDP and IR were generally below the observed values, indicating antagonism (Fig. 3A,B). This observation was confirmed by the results of Chou–Talalay median effect analysis (Fig. 3C,D).

### Cell growth after treatment

AHH-1 and VH10 cells were passaged for 21 and 30 days respectively after treatment and population doublings were recorded for controls, IR, selected doses of cDDP (0.2 µM for AHH-1 and 0.1 µM for VH10) and combined treatment. For AHH-1 (Fig. 4A), IR alone significantly (p < 0.01) and very largely (d = 5.22) inhibited cell proliferation over the whole period of observation. A similar result was observed for the combination treatment (p < 0.01, d = 4.01). CDDP alone had a weaker, but similarly persistent effect (p > 0.05, d = 1.63). No difference was observed between IR alone and the combined treatment (p > 0.05, d = 0.1)). A similar effect was observed in VH10 cells (Fig. 4B), however, the differences between treatments tended to decline with observation time, mainly because of decreasing proliferation of control cells. Combined treatment largely but insignificantly reduced cell growth as compared to IR alone (p > 0.05, d = 1.04).

### Cell death after treatment

Due to cell type difference in the main death pathway, the effect on cell death was determined 3 and 7 days after treatment by analysing caspase-3 positive AHH-1 cells (Fig. 4C) and beta galactosidase positive VH10 cells (Fig. 4D), see supplementary material for examples of results (Figs. 3, 4). In both cell types, cDDP alone had a significant and very large (AHH-1, p < 0.05, d = 1.99) and non-significant and large (VH10, p > 0.05, d = 1.3) effect on day 3 that declined on day 7 (AHH-1 p > 0.05, d = 0.23; VH10 p > 0.05, d = 1.59). Increase of cell death in the IR group was non-significant but very large on day 3 for AHH-1 (p > 0.05, d = 5.92) and decreased on day 7 although still very large (p > 0.05, d = 3.58). In VH10, more senescent cells were present on day 7 as compared to day 3. Except for AHH-1 cells on day 7, cell death after combined treatment was significantly and largely or very largely higher than after IR alone (AHH-1 3 days: p < 0.05, d = 1.09; VH10 3 days: p < 0.01, d = 2.29; 7 days: < 0.01, d = 1.06).

### Colony formation and sensitivity of cells to acute doses of IR after treatment

Long-term effects on colony formation were investigated 10 days after treatment. Colony formation of AHH-1 cells (Fig. 4E) insignificantly but very largely declined in all treatment groups with no difference between IR alone and combination groups (p > 0.05, d = 0.08). On the contrary, in VH10 fibroblasts (Fig. 4F), non-significant but very large increase in colony formation was observed in cDDP alone (p < 0.05, d = 1.93) and IR alone (p > 0.05, d = 3.73). The colony formation in the combination group was non-significantly but largely higher than the control (p > 0.05, d = 1.1).

To determine how each treatment would affect the subsequent radiosensitivity of surviving cells, clonogenic survival assays following acute exposure to IR were performed 20 days after treatment. AHH-1 cells treated with cDDP alone displayed a significant and very large decreased radiosensitivity (Fig. 4G, p > 0.01, d = 19.89). A similar but weaker effect was observed in the IR alone group (p < 0.01, d = 4.31). The radiosensitivity of cells from the combination group was non-significantly, but very largely reduced compared to control cells (p > 0.05, d = 3.1). It should be noted that the observed differences in radiosensitivity are largely driven by results from doses of 2 Gy and lower. There were no significant or large changes in the radiosensitivity of treated vs untreated VH10 cells (Fig. 4H).

### GammaH2AX foci after treatment

The levels of residual γ-H2AX foci were analysed 3 and 7 days after treatment (Fig. 5). Both the numbers of cells with foci (AHH-1: Fig. 5A and VH10: Fig. 5B) and the frequencies of foci per cell (AHH-1: Fig. 5C and VH10: Fig. 5D) were analysed.

Large interexperimental differences were observed and, consequently, none of the treatments induced a response that was significant. However, the effect size of some differences was large (Fig. 5A 7 days: IR alone vs combined treatment d = 1.01, Fig. 5B 3 days: control vs 0.1 µM cDDP d = 1.21; control vs IR alone d = 1.21, Fig. 5C 3 days: IR alone vs combined treatment d = 1.01, Fig. 5C 7 days: control vs combined treatment d = 1.27, Fig. 5D 3 days: control vs 0.1 µM cDDP d = 0.89, Fig. 5D 3 days: IR alone vs combined treatment d = 1.1, Fig. 5D 7 days: IR alone vs 0.1 µM cDDP d = 1.0) or very large (Fig. 5A 3 days: control vs IR alone d = 1.42, Fig. 5A 3 days: IR alone vs 0.2 µM cDDP d = 1.49, Fig. 5A 7 days: control vs combined treatment d = 1.45, Fig. 5B 3 days: control vs combined treatment d = 1.49, Fig. 5B 3 days: IR alone vs combined treatment d = 4.0, Fig. 5C 3 days: control vs IR alone d = 1.61, Fig. 5C 3 days: IR alone vs 0.2 µM cDDP, Fig. 5D 3 days: control vs IR alone d = 1.82, Fig. 5D 7 days: control vs 0.1 µM cDDP d = 1.87). Overall, for AHH-1, treatment increased the number of cells with foci. For VH10, there was an induction after single treatments at first, but later on it rather reduced that number, especially on day 7. A similar pattern was seen for frequency of foci per cell (Fig. 5C,D).

### Micronuclei, nuclear buds and giant nuclei after treatment

Micronuclei (MN) were scored in both cell types 3 and 7 days after treatment. Exemplary images are shown in Fig. 6A,C. In AHH1-1 cells all treatments induced a significant and very large (d = 15 and higher) frequency of MN after 3 days (Fig. 6B). The effect was still significant after 7 days, however its effect size was reduced (d < 10). In VH10 cells similar results were observed however they were not significant due to large interexperimental variation and the sizes of the effect were also lower (d < 3). No consistent difference in the effect was seen between IR alone and combined exposure. Giant nucleus (GN) formation was only detected, and therefore quantified, in AHH-1 cells (Fig. 6E). CDDP alone led to a significant (p < 0.01) and very large (d = 4.11) reduction of GN frequency on day 7 but not on day 3 (Fig. 6F). The strongest effect was induced by IR alone (3 days: p < 0.01, d = 25.72, 7 days: p < 0.01, d = 3.17). Combined treatment had a significantly lower effect than IR alone after 3 days (p < 0.01, d = 12.48) but the difference was no longer visible after 7 days (p > 0.05, d = 0.4).

### Stem cells markers after treatment

A proportion of therapy-induced polyploid giant cancer cells have been reported to survive and contribute to a cancer stem cell phenotype and tumour progression^23^. Therefore, gene expression of cancer and normal stem cells markers CD133, Nanog, Oct4, and Sox2 was analysed in AHH-1 cells in which giant cells were detected. Analysis was carried out 7, 19 and 21 days after treatment. Overall, the strongest effect was observed in cells exposed to IR alone and to combined treatment (Fig. 6G–J). Due to interexperimental variability none of the differences were statistically significant but both large and very large effects sizes were detected. CD133 showed the highest levels 7 days after treatment (Fig. 6G). Nanog, Oct4 and Sox2 were elevated later, on days 19 and 21 (Fig. 6H–J). There was no clear difference between IR alone and combined treatment.

## Discussion

The aim of this study was to determine the immediate and delayed effect of fractionated exposure to cDDP and IR given alone and in combination on two normal cell types. Cell growth was monitored during exposure and endpoints associated with carcinogenesis were assessed up to 21 days after treatment. The cell growth results demonstrate that cDDP does not enhance but rather weakens the effect of IR.

The result fits well with epidemiological observations suggesting that CT can reduce the risk of RT-associated SMN^12–14,24^. The observations, which are mainly related to breast cancer as SMN, are explained by hormonal changes due to CT-induced ovarian damage that also leads to premature menopause^14^. Obviously. this mechanism does not apply to our results which were achieved with in vitro cell systems. So how can our results be explained? Is the effect seen at the level of cell growth during treatment supported by post-treatment analyses of the cells?

Cell growth curves analysed after treatment grossly reflected the results observed during treatment. Cumulative population doubling of AHH-1 cells treated with 0.2 µM cDDP increased linearly with time with a slope somewhat lower than that of control cells. The growth curves of cells exposed to IR alone and combination of cDDP plus IR bent upwards indicating recovery (Fig. 4A). The level of apoptotic cells was highest in cells exposed to IR and combination both on day 3 and 7 after treatment but strongly declined between the day 3 and 7 (Fig. 4C) which is coherent with the process of recovery. Significantly more apoptosis was seen on day 3 in the combination group as compared to IR alone, an effect that was no longer detectable at day 7. Given the fact that apoptosis is an early response to DNA damage^25^, the elevated apoptosis frequency observed on day 3 post-treatment in the combination group could serve as explanation for the antagonistic effect of cDDP and IR. Proliferation of cells exposed to cDDP plus IR was higher than expected because slowly proliferating damaged cells were eliminated. This overkill effect may also explain the lack of difference between IR alone and IR plus cDDP in colony formation (Fig. 4E), the low level of giant cells on day 3 after treatment (Fig. 6F) and the lower γH2AX levels (Fig. 5A,C) at day 3 for combination compared to IR. However, it does not fit with the slight difference in radiosensitivity (Fig. 4G) or the elevated γH2AX levels in the combination group on day 7 after treatment (Fig. 5A,C). Actually, increased radioresistance and reduced residual damage would be expected if elimination of highly damaged cells took place during treatment. Nevertheless, the apoptosis results do suggest an overkill effect in the combination group that could serve as mechanistic explanation of the antagonism between cDDP and IR.

The analysis of delayed effects in VH10 cells and their comparison to AHH-1 is particularly interesting because, in contrast to AHH-1, the combination of lowest cDDP dose and IR resulted in an additive, not antagonistic response (Fig. 3D). Thus, the direction of delayed effects observed in both cell types exposed to combined treatment can be used as validation of the overkill hypothesis postulated for AHH-1 cells. If similar effects are seen in both cell types, then the overkill effect hypothesis does not hold. Similarly as in AHH-1 cells, cell growth curves of VH10 cells after treatment grossly reflected the results observed during treatment (Fig. 3B). However, the growth curves of control cells and those treated with cDDP saturated with time, while those of IR and combined treatment did not. The saturating curves can be explained by the aging of VH10 cells^22^ that is delayed in cells treated by IR and combination due to reduced proliferation during treatment. Cell senescence was highest in the combination group and increased between days 3 and 7 (Fig. 4D). This result particularly challenges overkill as the explanation for the antagonistic effect of cDDP and IR because the impact of combined treatment on cell doubling during treatment was additive and not antagonistic. Colony formation in cDDP alone and IR alone groups was highest and that in combination group was the same as in control cells (Fig. 4F). The radiosensitivity of cells from the different treatment groups did not differ (Fig. 4H) and the frequencies of delayed gammaH2AX foci (Fig. 5B and D) and micronuclei (Fig. 6D) were not consistently different from those observed in AHH-1 cells.

Taken together, the results of delayed effects do not provide a mechanistic explanation for the antagonistic effect between cDDP and IR. In an earlier study, we observed a lower level of cytogenetic damage in lymphocytes of gynaecological patients receiving CRT as compared to RT alone that was associated with enhanced level of apoptosis in the CRT arm^15^. We explained this result by an overkill effect but this explanation does not apply to the present results. If not by excessive cell killing, why does cDDP reduce the effect of IR?

CDDP affects cells in a multitude of ways that depend on external factors such as the level of oxygen^5,26^. At the DNA level cDDP binds at N7 sites of purine bases leading to the formation of platinum–DNA adducts (mainly intrastrand guanine–guanine crosslinks)^5^. Persistence of these platinum–DNA adducts can lead to stalled replication folk, inhibition of DNA damage repair and ultimately cell death^5,26^. There is no doubt that the combined application of cDDP and IR improves cancer cure^26^. However, results of in vitro experiments, mainly carried out with cancer cells treated with single doses of both compounds, yield controversial results with reported synergism^27^, additivity^28^ and subadditivity/antagonism^29^. The reason for these conflicting results are not known^18^. From the perspective of DNA lesions it was suggested that cDDP may potentiate the level of IR-induced DSB^30,31^ and inhibit or accelerate DNA repair depending on the concentration^32^. The potentiation of DSB formation by cDDP was only detected following treatment with very high doses of both agents and it was suggested that non-DSB cluster lesions may actually be responsible for any interaction between IR and cDDP^33^. On the other hand, due to its crosslinking activity cDDP stabilizes the DNA double helix and prevents the transformation of densely positioned SSB to DSB^34^. Obviously, the mechanisms of therapeutic gain from combining IR with cDDP is multifactorial and cannot only be reduced to the interaction at the level of DNA damage and repair.

The delayed manifestation of residual DNA damage and the formation of giant nuclei can be interpreted as indicators of pro-survival mechanisms which could lead to the initiation of carcinogenesis. Nanog, Oct 4 and Sox 2 are well known markers for both cancer and normal stem cells, and CD133 is a transmembrane reporter associated with hematopoietic stem cells, as well as several tumour types^35^. We analysed the expression of genes coding for these markers in the AHH-1 lymphoblasts as indicators of carcinogenic changes in the treated cells. Both IR alone and cDDP alone induced to some extent the expression of all stem cell markers. However, there was no evidence suggesting that the combined treatment consistently modulated this response. Hence, the analyses of stem cell markers are inconclusive regarding the mode of interaction between cDDP and IR.

A major problem of therapeutic application of chemotherapy drugs is multidrug resistance^26,36^. The repeated treatment with cDDP and IR in our study allowed assessing the possible acquisition of a resistance phenotype. However, as judged by the consistent linearity of growth curves during treatment, the resistance to cDDP or IR of AHH-1 and VH10 cells did not change during fractionation. An interesting observation was, however, the different relative sensitivity to both treatments following single and fractionated exposure. Following single exposure AHH-1 cells were more sensitive to IR and more resistant to cDDP than VH10 cells (supplementary material). Following fractionated treatment, the differential sensitivity to IR was retained but both cell types were equally sensitive to cDDP (Figure S2). While the mechanism of this result is not clear, it demonstrates the necessity of maintaining caution when transferring conclusions from studies with single to multiple exposures, the latter being more relevant to clinical exposure scenarios.

In conclusion, our results demonstrate that in a fractionated, concomitant in vitro exposure scenario cDDP reduces the inhibitory effect of IR on cell proliferation of normal cells and does not potentiate delayed effects resulting from accumulation of DNA damage. These results tie well with clinical results suggesting reduced risk of SMN following CRT. Although clinical results from breast SMN are explained by hormonal changes due to CT-induced ovarian damage, it is possible that antagonistic interaction of both factors on normal cells, as demonstrated in the present investigation, also play a role.

## Materials and methods

### Cells and cell culture

AHH-1 lymphoblasts (ATCC, USA, cat nr. CRL-8146, acquired in 2018, authenticated latest in 2022 by the presence of a reciprocal translocation between chromosomes 1 and 2) were cultured in RPMI-1640 medium with 25 mM HEPES, supplemented with 10% bovine calf serum (HyClone), 1% penicillin–streptomycin, 1% l-glutamine (200 mM), and 1% sodium pyruvate (100 mM), all from Sigma-Aldrich. AHH-1 lymphoblasts were passaged three times weekly in 75-cm^2^ culture flasks at a seeding density of 3.0 × 10^6^ cells during weekdays (Mondays, Wednesdays) and 2.0 × 10^6^ cells before weekends (Fridays). All cells were grown at 37 °C and 5% CO_2_. Primary normal human foreskin fibroblasts (VH10), donated in the early 2000s by prof. Leon Mullenders, Leiden University, Netherlands, were cultured in Dulbecco’s modified minimum essential medium (DMEM, Sigma-Aldrich) supplemented with 10% bovine calf serum (HyClone, Thermo Fisher Scientific, Waltham, MA, USA) and 1% penicillin–streptomycin (10.000 U penicillin and 10 mg streptomycin/ml, Sigma-Aldrich). The cells were put in culture by Enninga et al. in 1984 as described in^37^. Cell aliquots were frozen and thawed up at regular intervals. All experiments started with fibroblasts at passage 7 (P7) grown to 80% confluence before the start of each experiment. Cells were authenticated by their proliferation characteristics. VH10 fibroblasts were passaged weekly in 75-cm^2^ flasks at a seeding density of 3.5 × 10^5^ cells. Cells have been tested negative for mycoplasma infection during early phase of the experiments.

### Fractionated irIR

IrIR was carried out at room temperature using a ^137^Cs source (Scanditronix, Uppsala Sweden) at a dose rate of 0.35 Gy/min. Control samples were sham exposed. Cells were irradiated at 1 Gy/fraction based on an earlier study^20^. AHH-1 lymphoblasts received daily fractions each week (5 fractions per week, Monday–Friday) for a total of 2 weeks receiving a total dose of 10 Gy. VH10 fibroblasts were irradiated three times per week (Monday, Wednesday and Friday) for 3 weeks, receiving a total dose of 9 Gy (Fig. 1A).

### Fractionated cDDP treatment

CDDP (EMD Millipore, 232120, molar mass 300.05 g/mol) was reconstituted in 0.9% NaCl supplemented with 5% d-glucose and 5% d-mannitol (Sigma-Aldrich, Germany). Cells were treated with 0.1, 0.2, 0.4, 0.8, 1.7 and 3.3 μM of cDDP twice weekly, always on Monday and Wednesday, 4–6 h before irIR. AHH-1 lymphoblasts received a total of 4 treatments, while VH10 fibroblasts received 6 treatments (Fig. 1A).

The standard clinical dose of cDDP is 40 mg/m^2^^15^. Assuming that 1 kg of tissue has a volume of 1 l, 3.3 µM of cDDP corresponds to 40 mg/m^2^ of an adult male of 180 cm and 80 kg^38^.

### Trypan blue exclusion assay and population doubling

Cell concentration and viability were determined during the whole course of the experiment (treatment and recovery—see Fig. 1B) using the trypan blue exclusion assay as described by^39^ using an automated cell counter (Cell Countess, Invitrogen, UK). Cell growth was monitored by determining the population doubling using: PD = ln (Nt/N0)/ln2 where Nt is the cell concentration after harvesting cells, N0 is the cell concentration seeded (seeding density). Cell growth curves were created by plotting the cumulative increase in population doublings after each passage against time.

### Apoptosis analysis by caspase 3 detection in AHH-1 cells

Three and 7 days after treatment, AHH-1 cells were washed twice with PBS, and fixed in ice cold 70% ethanol at a cell density of 1 × 10^6^ cells per millilitre. Caspase 3 activity was measured using the FITC active caspase-3 apoptosis kit (BD Pharmingen, USA) according to the kit protocol. Flow cytometry analysis was carried out using Moxi GO II (ORFLO, USA) and analysis was done on FCS express.

### Senescence associated β-galactosidase (SA-β-gal) assay in VH10 cells

Six hours after the last fraction, VH10 fibroblasts were detached using trypsin and seeded in triplicates at 2000 cells/well in six-well plates. Three and 7 days after treatment, VH10 fibroblasts were washed twice, before fixation at room temperature for 5 min in 4% paraformaldehyde, followed by washing with PBS. Fixed VH10 cells were then incubated in 1 ml of staining solution containing 0.1% X-gal, 5 mM potassium ferrocyanide, 5 mM potassium ferricyanide, 150 mM magnesium chloride, and 40 mM citric acid/sodium phosphate solution pH 6.0. Cells were incubated in the dark, overnight at 37 °C in the absence of CO_2_ followed by washing with PBS. SA-β-gal cells were scored manually using a bright field microscope at 10× magnification.

### Colony forming assay

Colony forming assay for AHH-1 lymphoblasts was performed 10 days after the last IR fraction using the soft agar as described by Borowicz et al.^40^. Colony fixation and staining were carried out simultaneously using a 5% Giemsa solution containing 25% methanol, and colonies were counted manually. Colony forming assay on VH10 fibroblasts was carried out using the agarose overlay colony formation assay as described by Chandna et al.^41^. Colony fixation, staining, and counting were carried out as described above.

### Immunofluorescence assay for γH2AX foci

AHH-l lymphoblasts were fixed in 3% paraformaldehyde and 2% sucrose in PBS for 15 min 3 and 7 days after the last IR fraction, followed by PBS washes and immunostaining according to the protocol described by Sollazzo et al.^42^. VH10 fibroblasts were detached six hours after the last fraction of IR using trypsin and seeded at high density in duplicates on 22 × 22 mm coverslips (VWR International, Sweden), placed in six-well plates containing medium, and incubated at 37 °C for either 3 or 7 days. Immunostaining was carried out as described above.

Images of individual cells were captured using a fluorescent microscope with a 100× lens (Nikon Eclipse E800, Nikon, Tokyo, Japan), a CCD camera and the image analysis system ISIS (Metasystems, Althusheim, Germany). The selection of cells for image acquisition was random. Details of image acquisition are described elsewhere^42^. A modified macro written for ImageJ software^43^, version 1.43u (http://imagej.en.softonic.com/), was used to calculate the numbers of γH2AX foci. A total of 100 cells were analysed for each dose.

### Micronuclei, nuclear buds and giant nuclei

Micronuclei (MN), nuclear buds (NBD) and giant nuclei were scored on the same images as γH2AX foci as described elsewhere^20^. MN are small nuclear bodies lying close to but not connected to the main nucleus. NBD are also nuclear bodies connected to the nucleus via a stalk. MN and NBD frequency was scored in mononucleated using the criteria outlined by Fenech et al.^44^. Giant nuclei were identified by their area, and area ranged from 80 to 160 μm^2^ compared to normal nuclei with average nuclei area of 35 μm^2^ in the controls. Analysis of the nucleus area was possible with the help of Isis.

### Gene expression of stem cell markers by real time qPCR

RNA extraction was carried out using the E.Z.N.A Total RNA Kit I (Omega Bio-tek), 7, 19 and 21 days after the last fraction. cDNA was synthesized from 500 ng RNA using the high-capacity cDNA reverse transcription kit (Thermo Fisher Scientific) with random hexamer primers. Real time quantitative PCR was performed using the 5xHOT FIREPol^®^ EvaGreen^®^ qPCR Supermix (Solis Biodyne, Estonia) in duplicate on LightCycler^®^ 480, starting at 95 °C for 15 min, followed by 40 cycles of 95 °C for 15 s, 60 °C for 20 s, and 72 °C for 20 s. Relative gene expression was calculated using the 2^−ΔΔCt^ method as described in Livak et al.^45^. Primers towards CD133, Nanog, Oct4, Sox2 and 18S are given in Lundholm et al.^46^.

### Single dose IR and cDDP treatment

In order to check if the relative sensitivity of AHH-1 and VH10 cells to cDDP and IR is different following single and repeated exposure, MTT assay for cell metabolic activity was carried out after exposure to cDDP and clonogenic cell survival was measured after exposure to ionizing IR. The methodology and results are not directly relevant to the main aim of the study and are therefore presented as supplementary results in the supplementary material.

### Combination index

The total numbers of assessed population doublings were used to estimate the median effect to analyse the possible interaction between IR and cDDP. Relative values were calculated by normalizing treated samples (cDDP, IR and combination) to untreated controls. The combination index was determined using the CompuSyn program as described by Chou and Talalay^47,48^. To derive the combination index, the effect of IR only and cDDP only on cell growth at different doses was first analysed. Fractionation effects of IR only on cell growth at increasing IR doses per fraction was taken from Akuwudike et al.^20^ and normalized as described above. Combination analysis was carried out using the non-constant ratio^47,48^.

### Statistical analysis

Cell growth curves during treatment were fitted to a linear function Y = αd, where d is the day of treatment and α is the fitting coefficient. Cell growth curves after treatment were fitted to the linear-quadratic function Y = αd + βd^2^. α coefficients, corresponding to the slope of a curve, were compared by one-way ANOVA with Holm-Sidak post hoc test (SigmaStat ver 4.0). A p-value below 0.05 was considered significant. Effect sizes were calculated by Cohen’s effect size test^49^. The application of both the significance and effect size test to assess the validity of the results is based on recommendations of statisticians^50,51^. The results of the statistical tests are not included in the figures, but in supplementary tables. The reason for doing this is to avoid overcrowding that would make the figures difficult to read.

Survival curves of VH10 fibroblasts, re-irradiated 20 days after the last IR fraction were fitted using the linear quadratic equation S = e^−(αD+βD2)^ where D is the dose in Gy, α and β are fitting coefficients, while curves of AHH-1 lymphoblasts were fitted to a linear equation S = e^−αD^.

The expected relative total population doublings shown in Fig. 3A,B were calculated based on assumed additivity between IR and cDDP: the sum of observed percent points by which each treatment reduced the population doubling with reference to the control were subtracted from 100.

### Supplementary Information

Supplementary Information.

## Data Availability

Data supporting the results reported in the article are available upon request from the corresponding author.
