# Supplementary material for: Impact of fractionated cisplatin and radiation treatment on cell growth and accumulation of DNA damage in two normal cell types differing in origin
Source: Sci Rep. 2023 Sep 9;13:14891. doi: 10.1038/s41598-023-39409-7 (PMC10492820; doi:10.1038/s41598-023-39409-7)

Supplementary table S1. P values and respective effect sizes (Cohen´s d values) for treatment pairs contained in figures 2A - 2L. Only significant results are shown (except control vs 1.7 µM cDDP which was not significant). One way ANOVA was carried out for data sets included in each figure by all pairwise multiple comparison procedures with Holm-Sidak post-hoc test. Cohen´s effect size test was carried out for each pair of results (treatment 1 vs treatment 2). cDDP: cisplatin, IR: ionising radiation. cDDP doses are given in µM. Significant p values and large and very large effects are marked in colour according the key given in the right column of the table.

AHH1

Comparison		ANOVA	Cohen´s
**Treatment 1**	**Treatment 2**	**p**	**d**
Control	IR	<0.01	5.69
Control	1.7 cDDP	>0.05	1.17
Control	3.3 cDDP	<0.01	6.47
Control	0.2 cDDP+IR	<0.01	5.16
Control	0.4 cDDP+IR	<0.01	5.83
Control	0.8 cDDP+IR	<0.01	5.71
Control	1.7 cDDP+IR	<0.01	6.02
Control	3.3 cDDP+IR	<0.01	6.56
0.2 cDDP	IR	<0.01	6.75
0.2 cDDP	3.3 cDDP	<0.01	7.86
0.2 cDDP	0.2 cDDP+IR	<0.01	5.62
0.2 cDDP	0.4 cDDP+IR	<0.01	7.31
0.2 cDDP	0.8 cDDP+IR	<0.01	7.19
0.2 cDDP	1.7 cDDP+IR	<0.01	7.96
0.2 cDDP	3.3 cDDP+IR	<0.01	7.86
0.4 cDDP	IR	<0.01	11.57
0.4 cDDP	3.3 cDDP	<0.01	15.07
0.4 cDDP	0.2 cDDP+IR	<0.01	7.51
0.4 cDDP	0.4 cDDP+IR	<0.01	21.52
0.4 cDDP	0.8 cDDP+IR	<0.01	18.01
0.4 cDDP	1.7 cDDP+IR	<0.01	28.58
0.4 cDDP	3.3 cDDP+IR	<0.01	17.14
0.8 cDDP	IR	<0.01	11.67
0.8 cDDP	3.3 cDDP	<0.01	15.16
0.8 cDDP	0.2 cDDP+IR	<0.01	7.59
0.8 cDDP	0.4 cDDP+IR	<0.01	21.47
0.8 cDDP	0.8 cDDP+IR	<0.01	11.66
0.8 cDDP	1.7 cDDP+IR	<0.01	28.24
0.8 cDDP	3.3 cDDP+IR	<0.01	17.28
1.7 cDDP	IR	<0.01	9.36
1.7 cDDP	3.3 cDDP	<0.01	11.71
1.7 cDDP	0.2 cDDP+IR	<0.01	6.60
1.7 cDDP	0.4 cDDP+IR	<0.01	13.12
1.7 cDDP	0.8 cDDP+IR	<0.01	12.14
1.7 cDDP	1.7 cDDP+IR	<0.01	15.26
1.7 cDDP	3.3 cDDP+IR	<0.01	12.87

VH10

Comparison		ANOVA	Cohen´s
**Treatment 1**	**Treatment 2**	**p**	**d**
Control	IR	<0.05	3.94
Control	1.7 cDDP	>0.05	3.49
Control	3.3 cDDP	<0.01	3.46
Control	0.2 cDDP+IR	<0.05	4.34
Control	1.7 cDDP+IR	<0.01	2.72
Control	3.3 cDDP+IR	<0.01	4.94
0.2 cDDP	IR	<0.05	4.75
0.2 cDDP	3.3 cDDP	<0.01	3.67
0.2 cDDP	0.2 cDDP+IR	<0.05	5.33
0.2 cDDP	1.7 cDDP+IR	<0.01	2.90
0.2 cDDP	3.3 cDDP+IR	<0.01	6.01
0.4 cDDP	3.3 cDDP	<0.01	2.88
0.4 cDDP	1.7 cDDP+IR	<0.05	2.89
0.8 cDDP	3.3 cDDP	<0.05	2.89
0.8 cDDP	1.7 cDDP+IR	<0.05	2.13

Interpretation of p values

>0.05	non significant
<0.05	significant

Interpretation of Cohen´s d values

**d value**	**Effect**
>0.2-0.5	small
>0.5-0.8	medium
>0.8-1.3	large
>1.3	very large

Supplementary table S2. P values and respective effect sizes (Cohen´s d values) for selected treatment pairs contained in figures 4-6. Included are treatment pairs relevent for analysing the interacion of IR and cDDP. One way ANOVA was carried out for data sets included in a figure and, if relevant, the day of observation, by all pairwise multiple comparison procedures with Holm-Sidak post-hoc test. Cohen´s effect size test was carried out for each pair of results (treatment 1 vs treatment 2). cDDP: cisplatin, IR: ionising radiation. cDDP doses are given in µM. Significant p values and large and very large effects are marked in colour according the key given in the right column on page 2 of the table.

	**AHH-1**					**VH10**			
	**Comparison**		**ANOVA**	**Cohen´s**		**Comparison**		**ANOVA**	**Cohen´s**
**Fig 4A**	**Treatment 1**	**Treatment 2**	**p**	**d**	**Fig 4B**	**Treatment 1**	**Treatment 2**	**p**	**d**
	Control	0.2 cDDP	>0.05	1.63		Control	0.1 cDDP	>0.05	1.35
	Control	IR	<0.01	5.22		Control	IR	<0.01	2.88
	Control	0.2 cDDP + IR	<0.01	4.01		Control	0.1 cDDP+IR	<0.01	3.88
	IR	0.2 cDDP + IR	>0.05	0.10		IR	0.1 cDDP+IR	>0.05	1.04
	IR	0.2 cDDP	<0.05	2.8		IR	0.1 cDDP	>0.05	2.37
**Fig 4C**	**Treatment 1**	**Treatment 2**	**p**	**d**	**Fig 4D**	**Treatment 1**	**Treatment 2**	**p**	**d**
3 days	Control	0.2 cDDP	<0.05	1.99	3 days	Control	0.1 cDDP	>0.05	1.30
	Control	IR	>0.05	5.92		Control	IR	>0.05	3.58
	Control	0.2 cDDP + IR	<0.05	10.28		Control	0.1 cDDP+IR	<0.01	4.31
	IR	0.2 cDDP + IR	<0.05	1.09		IR	0.1 cDDP+IR	<0.01	2.29
	IR	0.2 cDDP	<0.01	3.78		IR	0.1 cDDP	>0.05	3.05
7 days	Control	0.2 cDDP	>0.05	0.23	7 days	Control	0.1 cDDP	>0.05	1.59
	Control	IR	>0.05	2.39		Control	IR	<0.01	21.44
	Control	<<	>0.05	3.28		Control	0.1 cDDP+IR	<0.01	17.47
	IR	0.2 cDDP + IR	>0.05	0.52		IR	0.1 cDDP+IR	<0.01	1.06
	IR	0.2 cDDP	>0.05	1.88		IR	0.1 cDDP	<0.01	21.16
**Fig 4E**	**Treatment 1**	**Treatment 2**	**p**	**d**	**Fig 4F**	**Treatment 1**	**Treatment 2**	**p**	**d**
	Control	0.2 cDDP	>0.05	3.47		Control	0.1 cDDP	>0.05	1.93
	Control	IR	>0.05	9.57		Control	IR	>0.05	3.73
	Control	0.2 cDDP + IR	>0.05	1.50		Control	0.1 cDDP+IR	>0.05	1.10
	IR	0.2 cDDP + IR	>0.05	0.08		IR	0.1 cDDP+IR	>0.05	2.75
	IR	0.2 cDDP	>0.05	3.64		IR	0.1 cDDP	>0.05	0.3
**Fig 4G**	**Treatment 1**	**Treatment 2**	**p**	**d**	**Fig 4H**	**Treatment 1**	**Treatment 2**	**p**	**d**
	Control	0.2 cDDP	<0.01	19.89		Control	0.1 cDDP	>0.05	0.49
	Control	IR	<0.01	4.32		Control	IR	>0.05	0.16
	Control	0.2 cDDP + IR	>0.05	3.41		Control	0.1 cDDP+IR	>0.05	0.35
	IR	0.2 cDDP + IR	>0.05	3.10		IR	0.1 cDDP+IR	>0.05	0.42
	IR	0.2 cDDP	>0.05	0.02		IR	0.1 cDDP	>0.05	0.53
**Fig 5A**	**Treatment 1**	**Treatment 2**	**p**	**d**	**Fig 5B**	**Treatment 1**	**Treatment 2**	**p**	**d**
3 days	Control	0.2 cDDP	>0.05	0.41	**3 days**	Control	0.1 cDDP	>0.05	1.21
	Control	IR	>0.05	1.42		Control	IR	>0.05	1.21
	Control	0.2 cDDP + IR	>0.05	0.19		Control	0.1 cDDP+IR	>0.05	1.49
	IR	0.2 cDDP + IR	>0.05	0.64		IR	0.1 cDDP+IR	>0.05	4.0
	IR	0.2 cDDP	>0.05	2.21		IR	0.1 cDDP	>0.05	0.38
7 days	Control	0.2 cDDP	>0.05	0.74	**7 days**	Control	0.1 cDDP	>0.05	0.75
	Control	IR	>0.05	0.27		Control	IR	>0.05	0.39
	Control	0.2 cDDP + IR	>0.05	1.45		Control	0.1 cDDP+IR	>0.05	0.36
	IR	0.2 cDDP + IR	>0.05	1.2		IR	0.1 cDDP+IR	>0.05	0.07
	IR	0.2 cDDP	>0.05	0.7		IR	0.1 cDDP	>0.05	0.37
**Fig 5C**	**Treatment 1**	**Treatment 2**	**p**	**d**	**Fig 5D**	**Treatment 1**	**Treatment 2**	**p**	**d**
3 days	Control	0.2 cDDP	>0.05	0.52	**3 days**	Control	0.1 cDDP	>0.05	0.89
	Control	IR	>0.05	1.61		Control	IR	>0.05	1.82
	Control	0.2 cDDP + IR	>0.05	0.32		Control	0.1 cDDP+IR	>0.05	0.15
	IR	0.2 cDDP + IR	>0.05	1.01		IR	0.1 cDDP+IR	>0.05	1.1
	IR	0.2 cDDP	>0.05	1.49		IR	0.1 cDDP	>0.05	0.39

7 days	Control	0.2 cDDP	>0.05	0.39
	Control	IR	>0.05	0.08
	Control	0.2 cDDP + IR	>0.05	1.27
	IR	0.2 cDDP + IR	>0.05	0.6
	IR	0.2 cDDP	>0.05	0.2

Fig 6B	Treatment 1	Treatment 2	p	d
3 days	Control	0.2 cDDP	<0.05	14.96
	Control	IR	<0.01	15.82
	Control	0.2 cDDP + IR	<0.01	17.05
	IR	0.2 cDDP + IR	>0.05	0.73
	IR	0.2 cDDP	<0.01	13.1
7 days	Control	0.2 cDDP	<0.05	5.92
	Control	IR	<0.01	9.03
	Control	0.2 cDDP + IR	<0.01	4.88
	IR	0.2 cDDP + IR	>0.05	0.97
	IR	0.2 cDDP	>0.05	3.1

Fig 6F	Treatment 1	Treatment 2	p	d
3 days	Control	0.2 cDDP	>0.05	0.30
	Control	IR	<0.01	25.72
	Control	0.2 cDDP + IR	<0.01	13.47
	IR	0.2 cDDP + IR	<0.01	12.48
	IR	0.2 cDDP	<0.01	25.83
7 days	Control	0.2 cDDP	<0.01	4.11
	Control	IR	<0.05	3.17
	Control	0.2 cDDP + IR	<0.05	2.84
	IR	0.2 cDDP + IR	>0.05	0.4
	IR	0.2 cDDP	<0.01	7.58

Fig 6G	Treatment 1	Treatment 2	p	d
7 days	Control	0.2 cDDP	>0.05	1.29
	Control	IR	>0.05	1.66
	Control	0.2 cDDP + IR	>0.05	1.67
	IR	0.2 cDDP + IR	>0.05	0.28
	IR	0.2 cDDP	>0.05	1.32
19 days	Control	0.2 cDDP	>0.05	1.01
	Control	IR	>0.05	0.26
	Control	0.2 cDDP + IR	>0.05	0.52
	IR	0.2 cDDP + IR	>0.05	0.31
	IR	0.2 cDDP	>0.05	0.57
21 days	Control	0.2 cDDP	>0.05	1.16
	Control	IR	>0.05	0.72
	Control	0.2 cDDP + IR	>0.05	0.40
	IR	0.2 cDDP + IR	>0.05	0.32
	IR	0.2 cDDP	>0.05	0.89

Fig 6H	Treatment 1	Treatment 2	p	d
7 days	Control	0.2 cDDP	>0.05	1.04
	Control	IR	>0.05	2.43
	Control	0.2 cDDP + IR	>0.05	0.69
	IR	0.2 cDDP + IR	>0.05	0.79
	IR	0.2 cDDP	>0.05	0.61
19 days	Control	0.2 cDDP	>0.05	0.81
	Control	IR	>0.05	0.89
	Control	0.2 cDDP + IR	>0.05	1.41
	IR	0.2 cDDP + IR	>0.05	0.03
	IR	0.2 cDDP	>0.05	0.54
21 days	Control	0.2 cDDP	>0.05	0.71
	Control	IR	>0.05	0.83
	Control	0.2 cDDP + IR	>0.05	3.65
	IR	0.2 cDDP + IR	>0.05	0.51

**7 days**	Control	0.1 cDDP	>0.05	1.87
	Control	IR	>0.05	0.33
	Control	0.1 cDDP+IR	>0.05	0.31
	IR	0.1 cDDP+IR	>0.05	0.07
	IR	0.1 cDDP	>0.05	1.1

Fig 6D	Treatment 1	Treatment 2	p	d
3 days	Control	0.1 cDDP	>0.05	2.10
	Control	IR	>0.05	2.59
	Control	0.1 cDDP+IR	>0.05	1.6
	IR	0.1 cDDP+IR	>0.05	0.13
	IR	0.1 cDDP	>0.05	1.62
7 days	Control	0.1 cDDP	>0.05	0.79
	Control	IR	>0.05	0.84
	Control	0.1 cDDP+IR	>0.05	2.02
	IR	0.1 cDDP+IR	>0.05	0.48
	IR	0.1 cDDP	>0.05	0.52

Interpretation of p values

>0.05	non significant
<0.05	significant

Interpretation of Cohen´s d values

d value	Effect
>0.2-0.5	small
>0.5-0.8	medium
>0.8-1.3	large
>1.3	very large

	IR	0.2 cDDP	>0.05	0.49

Fig 6I	Treatment 1	Treatment 2	p	d
7 days	Control	0.2 cDDP	>0.05	1.04
	Control	IR	>0.05	5.18
	Control	0.2 cDDP + IR	>0.05	0.89
	IR	0.2 cDDP + IR	>0.05	1.01
	IR	0.2 cDDP	>0.05	1.02
19 days	Control	0.2 cDDP	>0.05	0.74
	Control	IR	>0.05	0.93
	Control	0.2 cDDP + IR	>0.05	2.10
	IR	0.2 cDDP + IR	>0.05	0.05
	IR	0.2 cDDP	>0.05	0.59
21 days	Control	0.2 cDDP	>0.05	0.33
	Control	IR	>0.05	0.69
	Control	0.2 cDDP + IR	>0.05	2.43
	IR	0.2 cDDP + IR	>0.05	0.53
	IR	0.2 cDDP	>0.05	0.55

Fig 6J	Treatment 1	Treatment 2	p	d
7 days	Control	0.2 cDDP	>0.05	0.95
	Control	IR	>0.05	6.55
	Control	0.2 cDDP + IR	>0.05	4.01
	IR	0.2 cDDP + IR	>0.05	1.04
	IR	0.2 cDDP	>0.05	0.96
19 days	Control	0.2 cDDP	>0.05	0.72
	Control	IR	>0.05	1.03
	Control	0.2 cDDP + IR	>0.05	1.24
	IR	0.2 cDDP + IR	>0.05	0.74
	IR	0.2 cDDP	>0.05	0.62
21 days	Control	0.2 cDDP	>0.05	0.17
	Control	IR	>0.05	0.81
	Control	0.2 cDDP + IR	>0.05	32.08
	IR	0.2 cDDP + IR	>0.05	0.31
	IR	0.2 cDDP	>0.05	0.63

Supplementary results – differential sensitivity of AHH-1 and VH10 cells to cisplatin and ionizing radiation following single and fractionated exposure

In order to check if the sensitivity of AHH-1 and VH10 cells to cisplatin and ionizing radiation was different following single and repeated exposure, MTT assay for assessing cell metabolic activity was carried out after exposure to cisplatin and clonogenic cell survival was measured after exposure to ionizing radiation. The results were compared to cells growth measured during fractionated treatment with cisplatin and radiation.

Cisplatin sensitivity to single doses assessed by cell viability assay – materials and methods

Cisplatin (EMD Millipore, 232120, mol mass 300.05 g/mol) was reconstituted in 0.9% NaCl supplemented with 5% D-Glucose and 5% D-mannitol (Sigma-Aldrich, Germany). AHH-1 and VH10 cells were seeded in triplicates in a 96-well plate at cell densities $2x10^4$ and $2x10^3$cells per well, respectively, and treated with a single dose of cisplatin corresponding to 0, 0.00033, 0.0033, 0.033, 0.33, 3.3 and 33.3 µM. Cell viability was measured 24h later using the MTT assay as described in Mosman et al. [1]. Absorbance values were determined at a wavelength of 595 nm using a spectrophotometer. 3-4,5-Dimethyl-2-thiazolyl)-2,5-diphenyl-2H-tetrazolium bromide (MTT) was from Sigma-Aldrich.

Radiation sensitivity to single doses assessed by clonogenic survival assay – materials and methods

The radiosensitivity of AHH-1 lymphoblasts was assessed using the soft agarose colony-forming assay [2]. Cells were seeded at cell densities ranging from 1000 - 4000 cells in duplicates in six-well culture dishes, irradiated with single doses of 1, 2, 3, and 4 Gy (0.32 Gy/min, ^{137}Cs, Scanditronix, Uppsala Sweden) and incubated for seven days. The radiosensitivity of VH10 fibroblasts was assessed using the agarose overlay colony formation assay described above. Cells were seeded at a cell density of 5000 cells in 100-mm diameter cell culture dishes, and 24 hours later, growth media was discarded and replaced with 3 ml of agarose mixture as described above, followed by pouring 7 ml of growth media over the agarose overlay. The cells were irradiated at 2, 4, 6, 8 Gy (0.767 Gy/min, ^{137}Cs, Gammacell 40, Theratronix, Canada) and incubated for twenty-one days.

Radiation sensitivity to fractionated doses assessed by clonogenic survival assay – materials and methods

The sensitivity to fractionated doses of cisplatin and radiation was assessed by analysing population doublings. The methodology including the treatment doses is described in materials and methods of the article. Curves were fitted to linear functions as shown in Fig 2 of the article. In order to compare growth curve slopes of both cell types, the control slope of each cell type was set to 1 and slopes of treated cells were normalized to the control.

Results - single dose exposure

AHH-1 cells were more resistant to cisplatin than VH10 cells: the half maximal inhibitory concentration (IC_{50}) of cisplatin for cell viability measured with the MTT test was 1.75 µM and

0.642 µM for AHH-1 and VH10 cells (Fig S1A). In contrast, AHH-1 cells were more sensitive to radiation than VH10, with lethal dose at 50% (LD_{50}) values of 0.78 Gy and 2.04 Gy for AHH-1 and VH10 cells, respectively, as measured by clonogenic cells survival assays (Fig S1B).

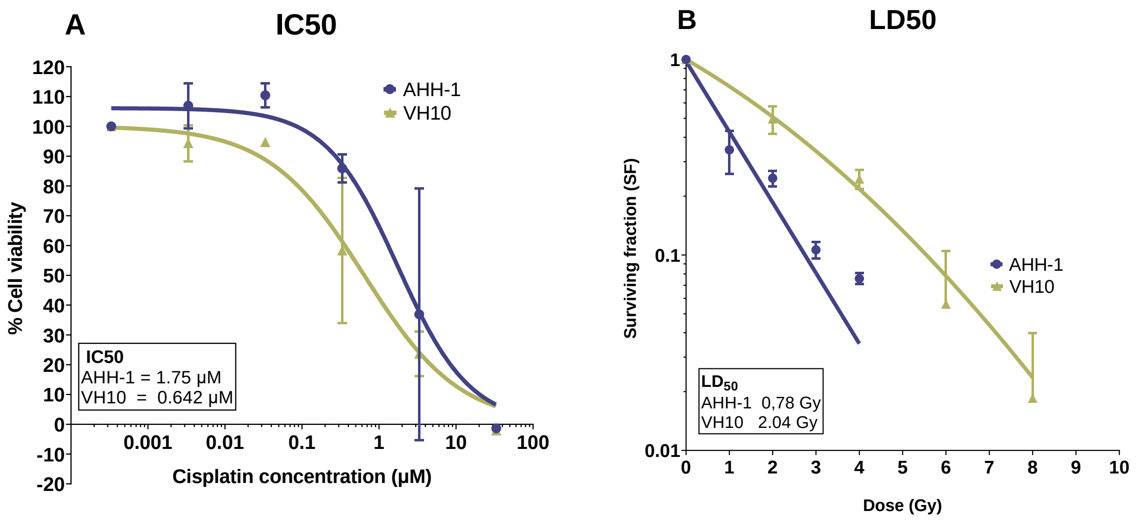

Figure S1. Panel A: cell viability of AHH-1 and VH10 cells exposed to single doses of cisplatin. Panel B: clonogenic cell survival following single dose exposure to ionizing radiation. IC50: half maximal inhibitory concentration, LD50: lethal dose at 50%. Error bars represent standard deviations.

Results - fractionated exposure

The sensitivity of AHH-1 and VH10 to cisplatin did not differ: while the slopes of the growth curves decreased with cisplatin dose, no consistent difference in the pattern was seen between both cell types. The differential sensitivity to radiation was the same as for single dose exposure, with AHH-1 cells showing a higher sensitivity (lower growth rate) than VH10 cells.

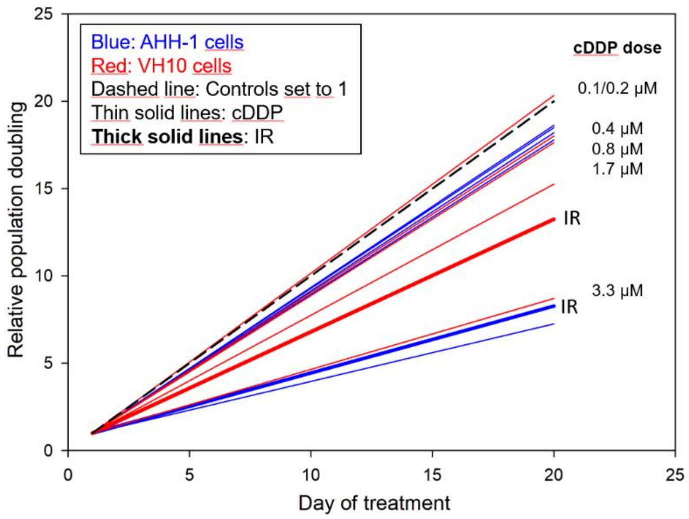

Figure S2. Relative population doublings representing growth curves of AHH-1 and VH10 cells exposed to fractionated doses of cisplatin (cDDP) and ionizing radiation (IR). Slopes of treated cells were normalised to the respective control that was set to 1. cDDP doses on the right margin illustrate the order of curves for both cell types.

Conclusion

The differential sensitivity of both cell types to cisplatin is influenced be the exposure pattern. The differential sensitivity to radiation is independent of the exposure pattern.

References

1 Mosmann, T. Rapid colorimetric assay for cellular growth and survival: Application to proliferation and cytotoxicity assays. *Journal of Immunological Methods* **65**, 55-63 (1983).

2 Sollazzo, A. *et al.* Interaction of low and high LET radiation in TK6 cells-mechanistic aspects and significance for radiation protection. *J. Radiol. Prot* **36**, 721-735 (2016).

Supplementary Figure S3. VH10 cells stained with X-gal against senescence associated β-galactosidase (SA-β-gal). A: irradiated VH10 cells. SA-β-gal positive cells are marked with red arrows, negative cells are markedd with blue arrows. B: control cells, all SA-β-gal negative. Cells were counter-stained with 1% orcein in 45% acetic acid for the purpose of taking images. Routine quantification of SA-β-gal-positive cells was performed under phase-contrast light microscopy without counterstaining.

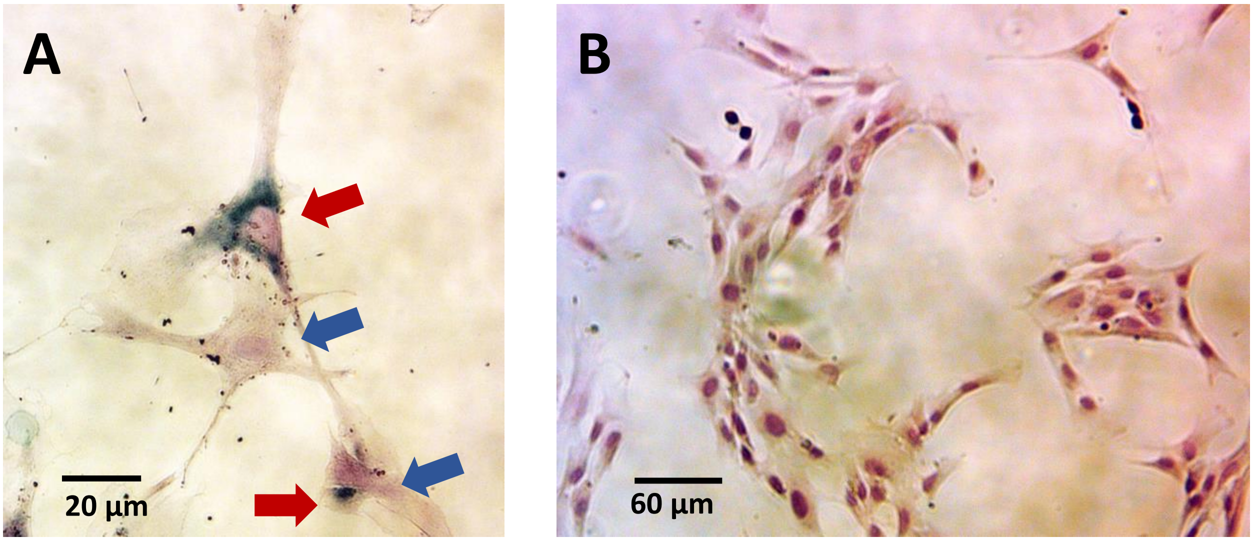

Supplement: Supplementary file 1 — Supplementary Information. [file 41598_2023_39409_MOESM1_ESM.pdf]
